# Mediastinal fat necrosis

**DOI:** 10.36416/1806-3756/e20260146

**Published:** 2026-05-22

**Authors:** Edson Marchiori, Bruno Hochhegger, Gláucia Zanetti

**Affiliations:** 1. Universidade Federal do Rio de Janeiro, Rio de Janeiro (RJ) Brasil.; 2. University of Florida, Gainesville (FL) USA.

A 35-year-old man complains of discomfort in the right lower pectoral region for a week, which has worsened in the last three days. He denies trauma and has no evidence of other comorbidities. Laboratory tests are unremarkable. Chest X-ray and electrocardiogram are normal. Pulmonary thromboembolism is suspected. Contrast-enhanced chest CT shows an ovoid fatty lesion on the right hemithorax, demarcated by a soft-tissue attenuation rim, compatible with mediastinal fat necrosis (MFN; Figure 1). A conservative approach is adopted, and the patient’s pain is relieved by nonsteroidal anti-inflammatory drugs.

**Figure 1:**
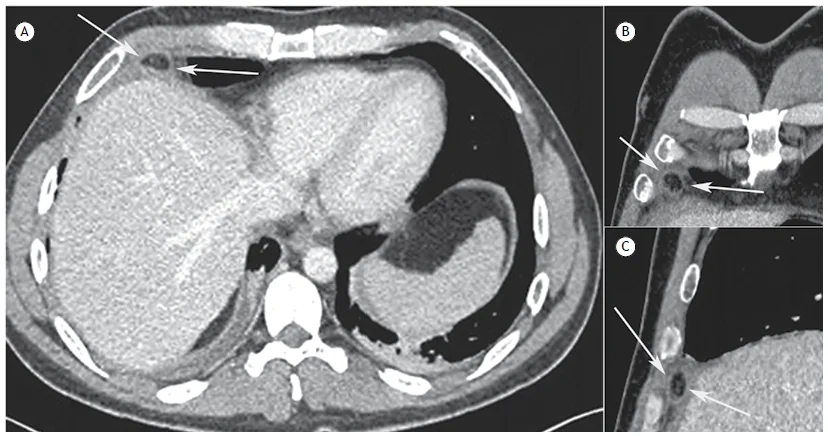
Contrast-enhanced chest CT images (soft-tissue window) obtained in the axial (A), coronal (B) and sagittal (C) planes showing an ovoid fatty lesion in the anterior right costophrenic space, demarcated by a soft-tissue attenuation rim (arrows) and a small right pleural effusion.

MFN, also known as epipericardial or pericardial fat necrosis, is a rare self-limited cause of acute chest pain and represents an inflammatory process that usually occurs in the juxtapericardial mediastinal fat and leads to encapsulated fat necrosis. MFN usually manifests as acute pleuritic chest pain in otherwise healthy patients and can mimic acute cardiopulmonary processes, such as myocardial infarction, pericarditis, and pulmonary embolism. Chest pain is characteristically ipsilateral to the lesion and is expected to subside gradually ceasing within several days. Electrocardiographic and laboratory findings are usually normal, but the C-reactive protein level may be elevated.^1^ ^-^ ^3^ The radiologist assumes a fundamental role in the diagnosis of this condition, because imaging examinations, especially chest CT and MRI, permit the detection, localization, and characterization of the lesion. Chest X-rays are frequently normal. CT can demonstrate an ovoid or rounded fatty lesion demarcated by a soft-tissue attenuation rim in the mediastinum, usually in the left juxtapericardial fat, associated with dense stranding of the surrounding mediastinal fat. A small ipsilateral pleural effusion is often present. Treatment is conservative, usually relying on nonsteroidal anti-inflammatory medications, with pain often resolving within a week. However, in severe cases or cases of diagnostic uncertainty a surgical approach may be indicated. ^1^ ^-^ ^3^

In conclusion, MFN should be considered in patients with unexplained chest pain and characteristic imaging findings. Chest CT is a valuable tool to confirm the diagnosis. Awareness among radiologists and emergency clinicians is essential for accurate diagnosis and avoidance of invasive interventions.

## References

[1] Marchiori E, Hochhegger B, Zanetti G (2024). Mediastinal fat necrosis-an overlooked cause of chest pain. J Bras Pneumol.

[2] Saliba T, Nevesny F, Rotzinger D (2025). Epipericardial fat necrosis, the overlooked culprit behind chest pain a case report. J Med Case Rep.

[3] Moreira BL, Chaves HL, Santana PRP, de Araújo NT, Imaeda CJ, da Silveira NSS (2021). Surgically treated mediastinal fat necrosis an exception to conservative treatment. Indian J Thorac Cardiovasc Surg.

